# Ability of Current Machine Learning Algorithms to Predict and Detect Hypoglycemia in Patients With Diabetes Mellitus: Meta-analysis

**DOI:** 10.2196/22458

**Published:** 2021-01-29

**Authors:** Satoru Kodama, Kazuya Fujihara, Haruka Shiozaki, Chika Horikawa, Mayuko Harada Yamada, Takaaki Sato, Yuta Yaguchi, Masahiko Yamamoto, Masaru Kitazawa, Midori Iwanaga, Yasuhiro Matsubayashi, Hirohito Sone

**Affiliations:** 1 Department of Prevention of Noncommunicable Diseases and Promotion of Health Checkup Niigata University Graduate School of Medical and Dental Sciences Niigata Japan; 2 Department of Hematology, Endocrinology and Metabolism Niigata University Graduate School of Medical and Dental Sciences Niigata Japan; 3 Department of Health and Nutrition, Faculty of Human Life Studies University of Niigata Prefecture Niigata Japan

**Keywords:** machine learning, hypoglycemia, meta-analysis

## Abstract

**Background:**

Machine learning (ML) algorithms have been widely introduced to diabetes research including those for the identification of hypoglycemia.

**Objective:**

The objective of this meta-analysis is to assess the current ability of ML algorithms to detect hypoglycemia (ie, alert to hypoglycemia coinciding with its symptoms) or predict hypoglycemia (ie, alert to hypoglycemia before its symptoms have occurred).

**Methods:**

Electronic literature searches (from January 1, 1950, to September 14, 2020) were conducted using the Dialog platform that covers 96 databases of peer-reviewed literature. Included studies had to train the ML algorithm in order to build a model to detect or predict hypoglycemia and test its performance. The set of 2 × 2 data (ie, number of true positives, false positives, true negatives, and false negatives) was pooled with a hierarchical summary receiver operating characteristic model.

**Results:**

A total of 33 studies (14 studies for detecting hypoglycemia and 19 studies for predicting hypoglycemia) were eligible. For detection of hypoglycemia, pooled estimates (95% CI) of sensitivity, specificity, positive likelihood ratio (PLR), and negative likelihood ratio (NLR) were 0.79 (0.75-0.83), 0.80 (0.64-0.91), 8.05 (4.79-13.51), and 0.18 (0.12-0.27), respectively. For prediction of hypoglycemia, pooled estimates (95% CI) were 0.80 (0.72-0.86) for sensitivity, 0.92 (0.87-0.96) for specificity, 10.42 (5.82-18.65) for PLR, and 0.22 (0.15-0.31) for NLR.

**Conclusions:**

Current ML algorithms have insufficient ability to detect ongoing hypoglycemia and considerate ability to predict impeding hypoglycemia in patients with diabetes mellitus using hypoglycemic drugs with regard to diagnostic tests in accordance with the Users’ Guide to Medical Literature (PLR should be ≥5 and NLR should be ≤0.2 for moderate reliability). However, it should be emphasized that the clinical applicability of these ML algorithms should be evaluated according to patients’ risk profiles such as for hypoglycemia and its associated complications (eg, arrhythmia, neuroglycopenia) as well as the average ability of the ML algorithms. Continued research is required to develop more accurate ML algorithms than those that currently exist and to enhance the feasibility of applying ML in clinical settings.

**Trial Registration:**

PROSPERO International Prospective Register of Systematic Reviews CRD42020163682; http://www.crd.york.ac.uk/PROSPERO/display_record.php?ID=CRD42020163682

## Introduction

Hypoglycemia is a major barrier to achieving the tight glycemic control in patients with diabetes mellitus (DM) that is required to delay the progression of late DM-related complications. Although many patients exhibit symptoms of hypoglycemia such as anxiety, heart palpitations, and confusion, a significant number have diminished ability to recognize these hypoglycemic symptoms [1,2], which is defined as “impaired awareness of hypoglycemia” [3]. This impaired awareness can lead to severe hypoglycemia, which is associated with seizures, coma, and death. Real-time glucose monitoring can help patients maintain optimal glycemic control while avoiding symptomatic or asymptomatic hypoglycemia [4]. However, the traditional monitoring method, intermittent glucose monitoring by finger stick, provides only a limited number of readings and is unlikely to detect hypoglycemia of a short duration. Continuous glucose monitoring (CGM) typically produces a reading every 5 minutes and can alert the patient to not only the occurrence of hypoglycemia but also impending hypoglycemia [5]. Accuracy of CGM has progressively improved, with overall measurement errors reduced by twofold than in the first commercially available CGM devices introduced in 2000 [5].

However, even if CGM advancements enabled patients to continuously track their subcutaneous glucose levels, the statistical disadvantage of the CGM data stream would remain as a major limitation. The autocorrelation of the CGM reading vanishes after 30 minutes, meaning that the projection of blood glucose levels more than 30 minutes ahead would be inaccurate [6]. This finding suggests that the algorithm for identifying hypoglycemia should consider a patient’s contextual information such as diet, physical activity, and medications (including insulin) as well as various features of the CGM trend arrow [7].

Machine learning (ML) algorithms have been widely introduced to diabetes research including those for identification of hypoglycemia. The growing use of mobile health (mHealth) apps, sensors, wearables, and other point-of-care devices, including CGM sensors for self-monitoring and management of DM, have made possible the generation of automated and continuous diabetes-related data and created the opportunity for applying ML to automated decision support systems [8]. Combining ML-based decision support systems with the abundance of generated data has the potential to identify hypoglycemia with greater accuracy.

Conventionally, ML has been applied to detect abnormalities in blood glucose levels using physiological parameters that are highly correlated with hypoglycemia (eg, changes in brain or cardiac electrical activities) [7]. Recently, in addition to the detection of hypoglycemia, ML-based decision support systems have been proposed for predicting hypoglycemia by using various historical data (eg, series of blood glucose data, other laboratory and demographic data, verbal data in medical records, or secure messages suggesting occurrence of hypoglycemic events) [8]. Despite many reports of ML algorithms for detecting or preventing hypoglycemia, their abilities have not been comprehensively or quantitatively assessed. This meta-analysis aims to assess the current ability of ML algorithms to detect or predict hypoglycemia in patients with DM.

## Methods

### Protocol Registration

The study protocol has been registered in the international prospective register of systematic reviews (PROSPERO; Registration ID: CRD42020163682).

### Literature Searches

We used Dialog to perform the electronic literature searches. The platform allows users to access and search 96 databases of peer-reviewed literature. Publication dates ranged from January 1, 1950, to September 14, 2020. Search terms consisted of 2 elements: (1) thesaurus and text words related to ML and (2) text terms related to hypoglycemia and thesaurus terms related to glucose monitoring or blood glucose. The use of the thesaurus term was limited to 2 databases: EMBASE (EMTREE terms) and MEDLINE (MeSH terms). The above 2 elements were combined using the BOOLEAN operator “AND” (Multimedia Appendix 1). Manual searches were added to review reference lists in relevant studies. If eligible studies were obtained from the reference lists, the reference lists in those studies were also examined. Manual searches were continued until no eligible study was found in the references lists.

Study inclusion criteria were (1) all participants had DM; (2) study endpoint was hypoglycemia; (3) researchers clarified that they originally trained the ML algorithm using training data to build a model for detecting or predicting hypoglycemia or the same researchers trained the ML algorithm in a previous study; (4) the model’s performance was tested using the test data; and (5) sensitivity and specificity for detection or prediction of hypoglycemia were presented or could be calculated.

Exclusion criteria were (1) an event-based study (ie, specificity could not be estimated because nonhypoglycemia data were not included in the test data), (2) a case study (ie, training and test data were derived from only 1 patient), and (3) a 2 × 2 contingency table consisting of the number of true positives, false positives, false negatives, and false positives could not be reproduced. If studies met all of the inclusion criteria but did not allow the reproduction of a 2 × 2 contingency table, we asked the corresponding author of these studies for the total number of test data sets (N-total) and events (N-hypo) so that we could reproduce the 2 × 2 table. If the same test data were shared by 2 or more eligible studies, we chose the most updated study in which the ML algorithm was considered to show the best performance.

The outcome of meta-analyses of diagnostic or prognostic tests is the extent of consistency between an index test and a reference standard. The index test is defined as a new test that is proposed when the method for perfectly diagnosing a target condition in all individuals does not exist or cannot be used. In this meta-analysis, it corresponded to an ML algorithm that classified the input data as either hypoglycemia or nonhypoglycemia. The reference standard is defined by a procedure that is considered the best available method for categorizing participants into having or not having a target condition. In this meta-analysis, it corresponded to methods for diagnosing hypoglycemia in clinical practice, which included measurement of glucose levels, the International Classification of Diseases (ICD) code for hypoglycemia, or experts’ subjective judgment. Evaluation item was the ability of ML algorithms to detect hypoglycemia (ie, alert to hypoglycemia coinciding with its symptoms) or the ability to predict hypoglycemia (ie, alert to hypoglycemia before its symptoms have occurred). In studies that assessed the ability for detection, data used for the index test (ie, the ML algorithm) and data used for a reference standard (ie, diagnosing hypoglycemia) had to be examined at the same time. In studies assessing predictive ability, the data input into the ML algorithm had to be examined before the diagnosis of hypoglycemia.

### Data Extraction

Data were extracted by two authors (SK and KF) Disagreements were resolved by discussion with a third author (HiS). We fundamentally selected 1 datum if there were 2 or more extractable data for a set of test data in an individual study. If an individual study tested 2 or more ML classification methods or 2 or more models for 1 ML classifier, we extracted the datum related to the classifier or model that the study proposed as the best. If 2 or more different results were presented for the same model depending on the prediction window or horizon, we extracted data on the result in relation to the longest prediction window or horizon.

The following study characteristics were extracted: first author, publication year, evaluated item (ie, detecting or predicting hypoglycemia), country, type of DM (ie, type 1 or type 2), number of study participants, N-total, N-hypo, mean or range of the patients’ age, time of day of hypoglycemic events, place of supposed hypoglycemic episode (ie, experimental, in-hospital, and out-of-hospital), ML algorithm used for classification into hypoglycemia and nonhypoglycemia, threshold of glucose level for hypoglycemia, method for diagnosing hypoglycemia, method for separating the database into training and test data, and profiling data that were input into ML algorithms for performance testing.

### Study Quality

To evaluate study quality, we used a revised tool to assess diagnostic accuracy of studies (QUADAS-2). The QUADAS-2 consists of 4 domains: selection of participants, index test, reference standard, and flow and timing. All 4 domains were used for assessment of risk of bias and the first 3 domains were used to assess the consensus of applicability. Each domain has 1 query in relation to the risk of bias or applicability consisting of 7 questions (Multimedia Appendix 2) [9]. A “Yes” answer was assigned 1 point.

### Data Synthesis

The ability of ML algorithms to detect hypoglycemia and predict hypoglycemia was independently assessed. For data that were used to test the model’s performance, the number of true positives, false positives, true negatives, and false negatives was calculated. The set of 4 data was pooled with a hierarchical summary receiver operating characteristic (HSROC) model [10]. Indicators for the model’s performance included sensitivity, specificity, positive likelihood ratio (PLR), which is calculated as (sensitivity/[1–specificity]), and negative likelihood ratio (NLR), which is calculated as ([1–sensitivity]/specificity). Study heterogeneity was assessed by calculating I^2^ values for PLR and NLR based on a multivariate random-effects meta-regression that considered within- and between-study correlations [11] and classifying them into quartiles (0% to <25%, low; 25% to <50%, low-to-moderate; 50% to <75%, moderate-to-high; >75%, high) [12]. Publication bias was statistically assessed as proposed by Deeks et al [13], wherein the logarithm of the diagnostic odds ratio is regressed against its corresponding inverse of the square root of the effective sample size.

Sensitivity analyses were added, and the analysis was limited to studies that shared similar characteristics in terms of the type of DM, time of day when hypoglycemia occurred, place of supposed hypoglycemic events, and the profiling data input into the ML algorithm. It is of note that at least four data sets are necessary to perform these sensitivity analyses because the HSROC model has 4 parameters: sensitivity, specificity, accuracy, and threshold. A two-sided *P*-value <.05 was considered statistically significant. All statistical analyses were performed using Stata 16 (StataCorp).

## Results

### Literature Searches

Multimedia Appendix 3 shows the flow chart of the procedure for selecting studies. Using prespecified search terms, 1226 articles were retrieved; 61 databases published at least one of the retrieved articles (Multimedia Appendix 4). Of these 1226 articles, 150 studies were selected for further review. Manual searches resulted in the addition of 32 studies for further review, making a total of 182 studies. Of these, 149 studies were subsequently excluded for various reasons. Specifically, 12 studies [14-25] presented insufficient data to allow reproduction of the 2 × 2 contingency table, although data on sensitivity and specificity were presented. We asked the authors of these studies to provide N-totals and N-hypos so that we could calculate the number of true positives, false positives, true negatives, and false negatives. However, only the author of 2 studies responded to our communication [15,25], and therefore the remaining 10 studies with insufficient data had to be excluded from the meta-analysis. Finally, 33 studies [15,20,25-55] were eligible.

### Data Extraction of Study Characteristics

Table 1 shows the summary of study characteristics. Of the 33 studies, 19 studies (58%) [26-31,33,35,36,38-42,44-47,54] predicted hypoglycemia, and the remaining 14 studies (42%) detected hypoglycemia [15,20,25,32,34,37,43,48-53,55]. As much as 25 of the 33 included studies (76%) [15,20,25-27,29,30,32,35,36,38,39,41-44,46-53,55] specified type 1 as the type of DM. Type 2 DM was specified in only 3 of these studies (9%) [28,31,45] and the remaining 5 studies [33,34,37,40,54] did not specify the type of DM.

Regarding the time of day when hypoglycemic events occurred, nocturnal hypoglycemia was the most frequently reported (14 studies of the 33 included studies; 42%) [15,20,26,30,32,35,36,41,44,49-53]). As to the place of the supposed hypoglycemic episode, 16 of the 19 studies that predicted hypoglycemia (84%) [26-30,35,36,38-42,44-47] supposed the event took place in an out-of-hospital setting. The remaining 3 studies (16%) [31,33,54] supposed hypoglycemia occurring in an in-hospital setting. Of the 14 studies that detected hypoglycemia, 11 studies (79%) [15,20,25,32,43,48-52,55] detected hypoglycemia in an experimental setting, where hypoglycemia was induced by a hypoglycemic clamp procedure. In 20 of the 33 included studies (61%) [15,20,25,27,29,31,32,35,36,38,41,43-45,49-52,54,55]), a hold-out method was used to separate the information in the database according to training and test data.

Multimedia Appendix 5 shows the profiling data input into the ML algorithm for testing its performance in detecting or predicting hypoglycemia. In the majority of the 19 studies for predicting hypoglycemia (13 studies; 68%) [26-30,35,36,38,40-42,46,47], historical CGM data were input into the ML algorithm while the remaining 6 studies (32%) [31,33,39,44,45,54] did not use CGM. Of the 14 studies that detected hypoglycemia using ML, 7 studies (50%) [20,25,32,49,50,52,55] used information from electroencephalograms (EEGs) and 4 studies (29%) [15,43,51,53] used results of electrocardiography (ECG).

**Table 1:** Study characteristics of the 33 included studies to assess the ability of machine learning to detect or predict hypoglycemia.

Study source	Assessment^a^	Country	Type of DM	Patients, n	N-total^b^	N-hypo^c^	Mean or range of age (years)	Time^d^	Place^e^	Machine learning	Threshold of Hypo^f^ (mmol/L)	Method of Hypo detection^g^	Method of separation^h^
Bertachi et al [26]	Pre^k^	Spain	T1D^m^	10	124	39	32	Noc^p^	Out^s^	SVM^v^	3.9	CGM^ll^	nCV^oo^
Dave et al [27]	Pre	USA	T1D	112	637,735	18,233	13	N/S	Out	RF^w^	3.9	CGM	HO^pp^
Elhadd et al [28]	Pre	Qatar	T2D^n^	13	3918	172	51	N/S	Out	XGBoost	Unclear	CGM	nCV
Marcus et al [29]	Pre	Israel	T1D	11	43,533	5264	18-39	N/S	Out	KRR^x^	3.9	CGM	HO
Mosquera-Lopez et al [30], Test 1	Pre	USA	T1D	10	117	17	34	Noc	Out	SVM	3.9	CGM	ExV
Mosquera-Lopez et al [30], Test 2	Pre	USA	T1D	20	2706	258	35	Noc	Out	SVM	3.9	CGM	ExV
Mueller et al [31]	Pre	USA	T2D	453,487	90,687	2580	66	N/S	In^t^	REFS	3.9	Blood/ICD	HO
Ngo et al [32]	Dec^l^	Australia	T1D	8	135	53	12-18	Noc	Exp	BNN^y^	3.9	Blood	HO
Ruan et al [33]	Pre	UK	N/S^o^	17,658	3276	703	66	N/S	In	XGBoost	3.9	Blood	nCV
Rubega et al [25]	Dec	Italy	T1D	34	2516	1258	55	N/S	Exp^u^	NN^z^	3.9	Blood	HO
Chen et al [34]	Dec	USA	N/S	No data	300	11	No data	N/S	In	LR^aa^	N/A^kk^	Experts^mm^	nCV
Guemes et al [35]	Pre	USA	T1D	6	55	6	40-60	Noc	Out	SVM	3.9	CGM	HO
Jensen et al [36]	Pre	Denmark	T1D	463	921	79	43	Noc	Out	LDA^bb^	3	Blood	HO
Jin et al [37]	Dec	USA	N/S	No data	4104	132	No data	N/S	In	SVM	N/A	ICD^nn^	nCV
Oviedo et al [38]	Pre	Spain	T1D	10	1447	420	41	Pos^q^	Out	SVM	3.9	CGM	HO
Reddy et al [39]	Pre	USA	T1D	55	90	29	33	Ex	Out	RF	3.9	Blood	ExV
Seo et al [40]	Pre	Korea	N/S	104	7052	412	52	Pos	Out	RF	3.9	CGM	nCV
Arthur et al [41]	Pre	USA	T1D	6	51	6	40-60	Noc	Out	ANN^cc^	3.9	CGM	HO
Toffanin et al [42]	Pre	Italy	T1D	20	7096	36	46	N/S	Out	I-MPC^dd^	3.9	CGM	ExV
Ling et al [43]	Dec	Australia	T1D	16	269	55	15	N/S	Exp	FNN^ee^	3.3	CGM	HO
Sampath et al [44], DIA^i^	Pre	Ukraine	T1D	34	150	40	18-65	Noc	Out	RA	3.9	Blood	HO
Sampath et al [44], Child^j^	Pre	Ukraine	T1D	179	476	222	3-16	Noc	Out	RA^ff^	3.9	Blood	ExV
Sudharsan et al [45]	Pre	USA	T2D	Unclear	839	428	No data	N/S	Out	RF	3.9	Blood	HO
Eljil [46]	Pre	UAE	T1D	10	667	100	25	N/S	Out	BAG^gg^	3.3	CGM	nCV
Plis et al [47]	Pre	USA	T1D	2	5816	152	No data	N/S	Out	SVM	3.9	CGM	ExV
Jensen et al [48]	Dec	Denmark	T1D	10	1267	160	44	N/S	Exp	SEPCOR^hh^	3.9	Blood	LOO^qq^
Jensen et al [48]	Dec	Denmark	T1D	10	1267	160	44	N/S	Exp	+ SVM	3.9	Blood	LOO
Nguyen et al [49]	Dec	Australia	T1D	5	144	76	12-18	Noc	Exp	FNN	3.3	CGM	HO
Nguyen et al [50]	Dec	Australia	T1D	5	44	20	12-18	Noc	Exp	ANN	3.3	CGM	HO
Nuryani et al [51]	Dec	Australia	T1D	5	575	133	16	Noc	Exp	PSO^ii^ + SVM	Unclear	CGM	HO
Chan et al [15]	Dec	Australia	T1D	16	100	52	15	Noc	Exp	FNN	3.3	CGM	HO
Ling et al [52]	Dec	Australia	T1D	5	27	8	16	Noc	Exp	Fuzzy SVM	3.3	CGM	HO
Nguyen and Jones [20]	Dec	Australia	T1D	6	79	27	12-18	Noc	Exp	BNN	3.3	Blood	HO
Skladnev et al [53]	Dec	Australia	T1D	52	52	11	16	Noc	In	FNN	3.9	Blood	ExV
Zhang et al [54]	Pre	USA	N/S	1004	1114	556	No data	N/S	In	DT^jj^	3.3	CGM	HO
Iaione and Marques [55]	Dec	Brazil	T1D	8	1990	995	35	Mor^r^	Exp	ANN	3.3	Blood	HO

^a^Ability for which the machine learning algorithm was assessed.

^b^N-total: total number of data included in test data.

^c^N-hypo: total number of hypoglycemic episodes included in the test data.

^d^Time of day when hypoglycemia occurred.

^e^Place of supposed hypoglycemic episode.

^f^Threshold of glucose level that was used to diagnose hypoglycemia.

^g^Method for separating training and test data.

^h^Method used for diagnosing hypoglycemia.

^i^DIA: DIAdvisor.

^j^Child: ChildrenData.

^k^Pre: predicting hypoglycemia.

^l^Dec: detecting hypoglycemia.

^m^T1D: type 1 diabetes mellitus.

^n^T2D: type 2 diabetes mellitus.

^o^N/S: not specified.

^p^NOC: nocturnal hypoglycemia.

^q^Pos: postprandial.

^r^Mor: hypoglycemia during morning.

^s^Out: out-of-hospital setting.

^t^In: in-hospital setting.

^u^Exp: experimental setting (ie, hypoglycemia is induced by injection of insulin. Exercise or drug intervention is included in out of hospital setting).

^v^SVM: support vector machine.

^w^RF: random forest.

^x^KRR: Kernel Ridge Regression.

^y^BNN: Bayesian neural network.

^z^NN: neural network.

^aa^LR: logistic regression.

^bb^LDA: linear discriminant analysis.

^cc^ANN: artificial neural network.

^dd^I-MPC: individual model-based predictive control.

^ee^FNN: fuzzy neural network.

^ff^RA: ranking aggregation algorithms.

^gg^BAG: bagging (bootstrap aggregating).

^hh^SEPCOR: separability and correlation analysis.

^ii^PSO: particle swarm optimization.

^jj^DT: decision tree.

^kk^N/A: Not applicable.

^ll^CGM: continuous glucose monitoring.

^mm^Experts’ subjective judgment.

^nn^ICD: International Classification of Diseases.

^oo^nCV: n-fold cross-validation.

^pp^HO: hold-out method.

^qq^LOO: leave-one-out cross-validation.

### Assessment of Study Quality

Multimedia Appendix 6 shows the results of study quality assessments using QUADAS-2. Mean score (SD) was 5.6 (1.1), which corresponded to 80% of full marks (=7). The applicability of the reference test was evaluated to be low in 61% of the 33 included studies (20 studies) because hypoglycemia was not diagnosed by measuring blood glucose levels or ICD codes but by CGM (ie, glucose levels in blood are indirectly estimated from those in interstitial tissue) (19 studies) [15,26-30,35,38,40-43,46,47,49-52,54] or experts’ subjective judgement (1 study) [34]. The 2 factors were mainly responsible for lowering the study quality. We considered that the threshold of hypoglycemia in the index test was not specified in 7 studies, which used the cross-validation method [26,28,33,34,37,40,46], and 1 study, which used the leave-one-out method to separate test data from training data [48].

### Data Synthesis

#### Ability for Detection of Hypoglycemia Using ML Algorithms

**Figure 1:**
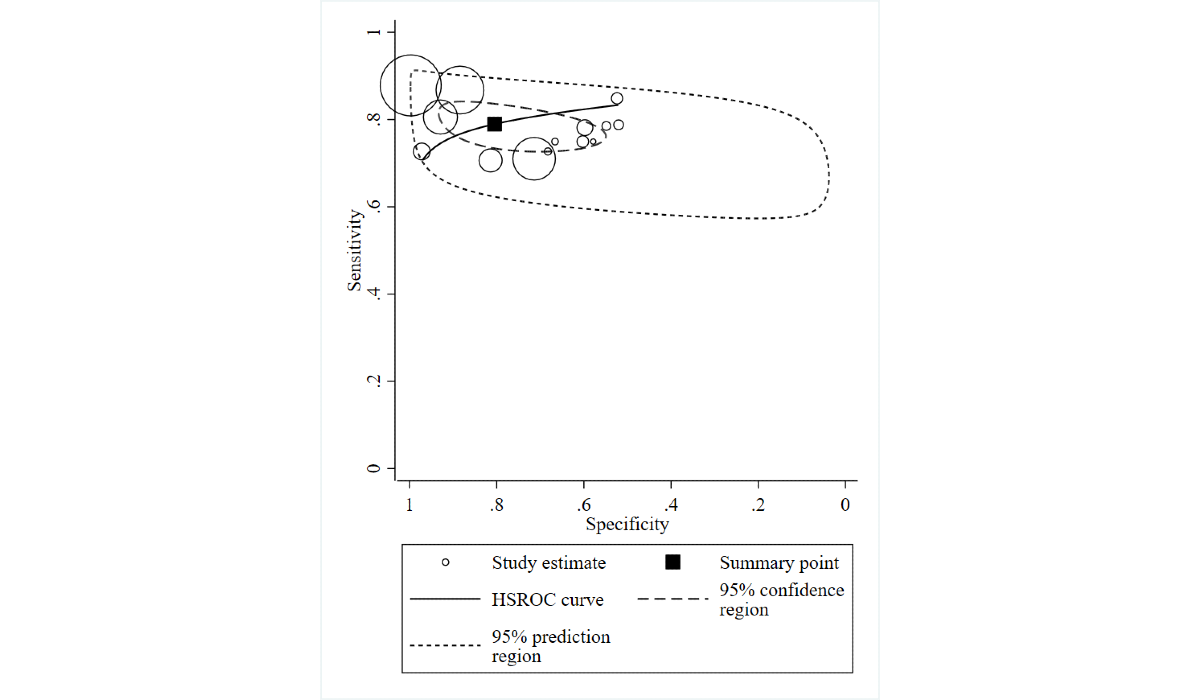
Hierarchical summary receiver-operating characteristic (HSROC) curve for detection of hypoglycemia using machine learning algorithms. Circles indicate study-specific sensitivity and specificity for each of the 14 included studies. The size of each circle is proportional to study sample size. The pooled point estimates of sensitivity and specificity are plotted in a filled square.

Figure 1 shows the HSROC curve and pooled estimates of sensitivity and specificity based on the 14 studies that assessed the ability of the ML algorithm to detect hypoglycemia. The pooled estimates (95% CI) were 0.79 (0.75-0.83) for sensitivity and 0.80 (0.64-0.91) for specificity. The pooled estimates (95% CI) of PLR and NLR were 2.20 (1.46-3.32) and 0.37 (0.28-0.49), respectively. Between-study heterogeneity expressed as I^2^ was high both for PLR (98%; 95% CI 95%-99%) and NLR (80%; 95% CI 50%-90%). Statistically significant publication bias was detected (*P*=.15).

We conducted several sensitivity analyses using a portion of the above 14 studies that had 1 study characteristic in common. It was not apparent that any of the sensitivity analyses showed results different from the overall analysis. Limiting the analyses to 12 studies [15,20,25,32,43,48-53,55] that specified type 1 as the DM type, pooled sensitivity, specificity, PLR, and NLR were 0.78 (95% CI 0.73-0.82), 0.71 (95% CI 0.60-0.79), 2.65 (95% CI 1.88-3.72), and 0.26 (95% CI 0.19-0.36), respectively. When analyses were limited to the 7 studies that detected nocturnal hypoglycemia using ML algorithms [15,20,49-53], the pooled estimates (95% CI) were 0.75 (0.70-0.80) for sensitivity, 0.65 (0.55-0.74) for specificity, 2.14 (1.67-2.76) for PLR, and 0.38 (0.30-0.48) for NLR. With analyses of the 11 studies that detected hypoglycemia in an experimental setting, pooled sensitivity, specificity, PLR, and NLR were 0.78 (95% CI 0.73-0.82), 0.71 (95% CI 0.60-0.80), 2.66 (95% CI 1.84-3.85), and 0.31 (0.24-0.41), respectively. The pooled estimate (95% CI) was 0.78 (0.71-0.84) for sensitivity, 0.67 (0.55-0.77) for specificity, 2.39 (1.63-3.50) for PLR, and 0.33 (0.22-0.48) for NLR when the analysis was limited to 7 studies that used EEG abnormalities for detecting hypoglycemia. These estimations were similar when limited to 4 studies that used ECG abnormalities for detection of hypoglycemia: pooled estimate (95% CI) was 0.76 (0.67-0.82) for sensitivity; 0.67 (0.54-0.78) for specificity; 2.31 (1.65-3.23) for PLR; and 0.36 (0.28-0.47) for NLR.

#### Ability to Predict Hypoglycemia Using ML Algorithms

**Figure 2:**
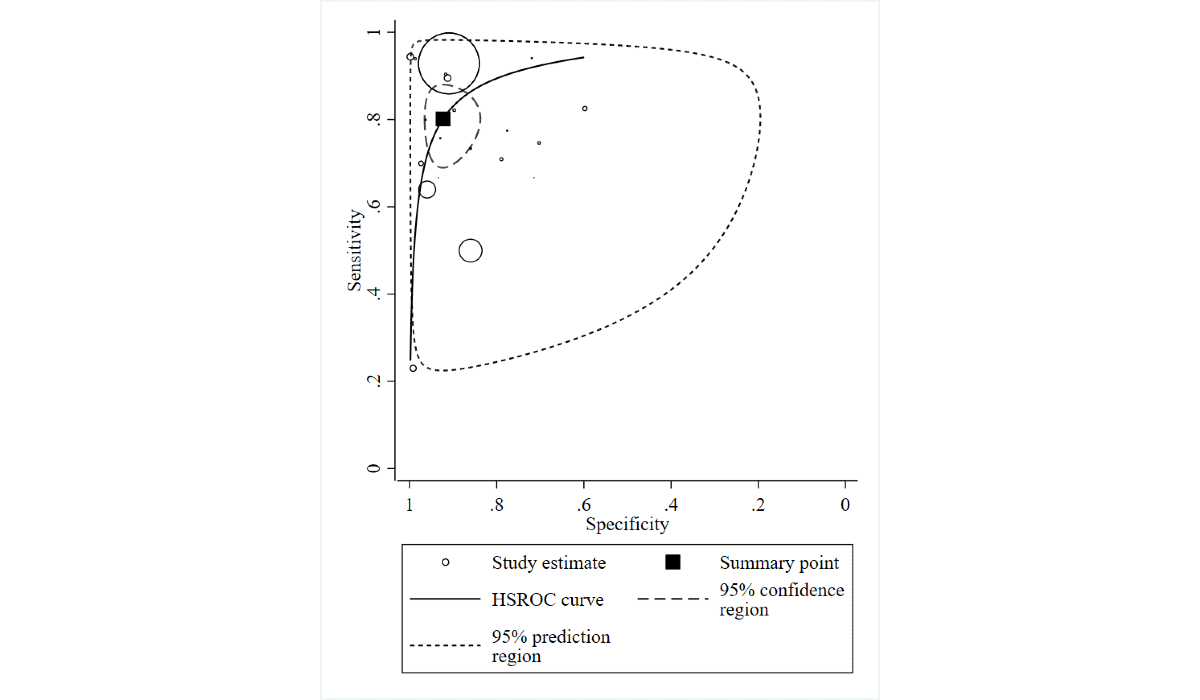
Hierarchical summary receiver-operating characteristic (HSROC) curve for prediction of hypoglycemia using machine learning algorithms. Circles indicate study-specific sensitivity and specificity for each of the 19 included studies. The size of each circle is proportional to study sample size. The pooled point estimates of sensitivity and specificity are plotted in a filled square.

Figure 2 shows the HSROC curve for predicting hypoglycemia based on the 19 studies that assessed the predictive ability for hypoglycemia. The point estimates (95% CI) were 0.80 (0.72-0.86) for sensitivity, 0.92 (0.87-0.96) for specificity, 10.42 (5.82-18.65) for PLR, and 0.22 (0.15-0.31) for NLR. Extremely high between-study heterogeneity was observed for both PLR (I^2^ [95% CI] 100% [100%-100%]) and NLR (I^2^ [95% CI] 99% [98%-100%]). Publication bias was not statistically significant (*P*=.68).

When the analyses were limited to 13 studies that specified type 1 as the DM type [26,27,29,30,35,36,38,39,41,42,44,46,47], the pooled estimates (95% CI) were 0.77 (0.67-0.85) for sensitivity, 0.92 (0.84-0.96) for specificity, 9.82 (4.58-21.04) for PLR, and 0.25 (0.16-0.38) for NLR. In the analyses of 7 studies that specified night as the time of hypoglycemic events [26,30,31,35,36,41,44], the predictive ability was low compared with that of the overall analysis—pooled estimate (95% CI): 0.74 (0.65-0.82) for sensitivity, 0.81 (0.72-0.88) for specificity, 3.98 (2.64-6.00) for PLR, and 0.31 (0.23-0.43) for NLR. Relatively high sensitivity and low NLR were observed in the 13 studies that used CGM historical data for predicting hypoglycemia—pooled estimate (95% CI): 0.82 (0.71-0.90) for sensitivity, 0.92 (0.83-0.97) for specificity, 10.41 (4.52-24.01) for PLR, and 0.19 (0.12-0.32) for NLR—compared with 6 studies that did not use CGM—pooled estimate (95% CI): 0.76 (0.66-0.84) for sensitivity, 0.92 (0.88-0.95) for specificity, 10.14 (6.13-16.77) for PLR, and 0.26 (0.17-0.38) for NLR). After excluding 3 studies [31,33,54] that showed that the supposed hypoglycemic events occurred in-hospital, the pooled estimates (95% CI) of the 16 studies with such events occurring in an out-of-hospital setting were 0.82 (0.74-0.88) for sensitivity, 0.92 (0.85-0.96) for specificity, 10.58 (5.44-20.55) for PLR, and 0.20 (0.13-0.39) for NLR.

## Discussion

### Principal Findings

Overall, the PLR and NLR of ML algorithms for detecting hypoglycemia were 4.05 and 0.26, respectively. These estimates were almost unchanged throughout several sensitivity analyses that were limited to studies that shared 1 characteristic in common. According to the Users’ Guide to Medical Literature with regard to diagnostic tests [56], the PLR should be 5 or more to moderately increase the probability of persons having or developing a disease and the NLR should be 0.2 or less to moderately decrease the probability of having or developing a disease after taking the index test. In summary, the current ML algorithms had insufficient ability to detect the occurrence of hypoglycemia. However, that would not mean that ECG or EEG monitoring in combination with ML, which was the case with 79% (11/14) of the included studies, was useless in detecting hypoglycemia. For example, for patients with both DM and high cardiovascular risk, in particular, those who are vulnerable to cardiac arrhythmias, using ECGs for detecting hypoglycemia is useful considering that a hypoglycemia-induced arrhythmia could contribute to increased cardiovascular mortality [57]. Similarly, for patients with repeated episodes of hypoglycemia, the combination of ML and EEG was indicated to be beneficial to prevent hypoglycemia-induced neuroglycopenia resulting in cognitive impairment and ultimately death, because blood glucose levels alone do not appear to predict that condition [58]. Thus, the clinical applicability of these devices should be evaluated by the individual’s risk of hypoglycemia and its related arrhythmia and neuroglycopenia as well as the overall ability of algorithms for ML.

The overall sensitivity, specificity, PLR, and NLR for predicting hypoglycemia were 0.80, 0.92. 10.42, and 0.22, respectively. Applying the above described guidelines for diagnostic tests to these results, it is worth considering the use of current ML algorithms as a tool for alerting patients to impending hypoglycemic events. In addition, it is considered that a test with a PLR over 10 has a particularly strong power to alter posttest probability of the targeted disease compared with pretest probability [56]. If a positive test result were to be received, patients with DM who are administered hypoglycemic treatments would be strongly recommended to pay more attention to the possibility of impeding hypoglycemic events than they would before receiving the predictive test for hypoglycemia. However, considering that the PLR and NLR values indicate relative risk (ie, risk of disease at posttest compared with that at pretest), the accuracy of predictive ability depends on patients’ risk of hypoglycemia in daily life. For example, even a less than 10% false-positive rate (8% in this meta-analysis) may be acceptable in patients at high risk of hypoglycemia but not in low-risk individuals due to too frequent false alarms. In such a case, there is fear that these patients will ignore the alarms and therefore miss the opportunity to take corrective action when the alarm is indeed true [59]. It is emphasized that the utility of ML algorithm depends on the extent of the patient’s risk of hypoglycemia. In addition, as indicated in the “Results” section, there was high between-study heterogeneity among studies. Specifically, when limiting analyses to the studies that predicted nocturnal hypoglycemia, the predictive ability was insufficient (pooled estimate: 3.98 for PLR; 0.31 for NLR). Considering that nocturnal hypoglycemia is the most common type of hypoglycemia among all hypoglycemic episodes [60], continued research is needed for further development of ML algorithms to predict hypoglycemia.

Several limitations of this meta-analysis should be addressed. First, the principal major limitation is the pooling of studies among which there was much variability in the type of DM, profiling data for detecting or predicting hypoglycemia, time of day when hypoglycemic events occurred, setting of supposed hypoglycemic events, and ML classification methods. In particular, although the ability for predicting hypoglycemia depended largely on the ML classification methods [33], this meta-analysis did not consider the difference in the test performance among various ML methods. Instead, the meta-analysis focused on ML’s comprehensive ability across studies using data in relation to the best model in each study, if 2 or more models existed, rather than comparisons among 2 or more models within 1 study. Given that generalization of evidence is among the most important roles in all meta-analyses, the issue of the variation in ML methods, in particular, the difference between old and new ML techniques, might be beyond the scope of this meta-analysis. Nevertheless, it should be emphasized that successful application of ML lies in the correct understanding of the advantages and disadvantages of different ML methods. Second, only 3 studies exclusively targeted patients with type 2 DM. With the increasing use of insulin to treat type 2 DM in the elderly, the prevalence of hypoglycemia is likely to escalate. In addition, the response to hypoglycemia is different between type 1 and type 2 DM [61]. Future studies should aim to develop and validate ML algorithms for detecting or predicting hypoglycemia in type 2 DM. Third, in most of the included studies, the ML classification models were developed in an experimental setting or by using previously recorded data as training and testing data instead of live data. Future studies need to train and test the algorithm on data from DM patients in everyday clinical practice to determine feasibility.

### Conclusion

Overall, current ML algorithms have insufficient ability to detect ongoing hypoglycemia and considerable ability to predict hypoglycemia in patients with DM receiving hypoglycemic treatments. However, the clinical applicability of these ML algorithms should be evaluated according to patients’ risk profiles such as for hypoglycemia and its associated complications (eg, arrhythmia, neuroglycopenia) as well as the average ability of the ML algorithm. Continued research is required to further develop ML algorithms to enhance their feasibility, considering the inaccuracy of CGM in the hypoglycemic range, the increased prevalence of hypoglycemia in the elderly, and increasing evidence for the effectiveness of tight glycemic control in preventing microvascular complications [62].

## Appendix

Multimedia Appendix 1 Search strategy in this meta-analysis.

Multimedia Appendix 2 Study quality assessment using the quality assessment of diagnostic accuracy studies (QUADUS-2).

Multimedia Appendix 3 Study flow in this meta-analysis.

Multimedia Appendix 4 Databases which published articles that were retrieved by the search terms (see Appendix 1).

Multimedia Appendix 5 Profiling data input into ML algorithm for testing its performance.

Multimedia Appendix 6 Results of assessing study quality using revised tool for the quality assessment of diagnostic accuracy studies (QUADUS-2). The criterion corresponding to each domain (D) and signaling question (SQ) is indicated in Appendix 2.

## Abbreviations

CGM: continuous glucose monitoring
DM: diabetes mellitus
HSROC: hierarchical summary receiver operating characteristic
ICD: International Classification of Diseases
ML: machine learning
N-hypo: total number of events
NLR: negative likelihood ratio
N-total: total number of test data sets
PLR: positive likelihood ratio
