# Supplementary material for: Ability of Current Machine Learning Algorithms to Predict and Detect Hypoglycemia in Patients With Diabetes Mellitus: Meta-analysis
Source: JMIR Diabetes. 2021 Jan 29;6(1):e22458. doi: 10.2196/22458 (PMC7880810; doi:10.2196/22458)
Supplement: Multimedia Appendix 1 [file diabetes_v6i1e22458_app1.docx]

S1 (EMB.EXACT.EXPLODE("artificial intelligence") OR EMB.EXACT.EXPLODE("machine learning")) OR (MESH.EXACT.EXPLODE("Machine Learning") OR MESH.EXACT("Artificial Intelligence")) OR ((learning NEAR/1 machine[*1]) OR "machine intelligence" OR "artificial intelligence")

S2 (MESH.EXACT("Blood Glucose Self-Monitoring")) OR (EMB.EXACT("blood glucose monitoring")) OR (MESH.EXACT("Blood Glucose -- analysis") OR MESH.EXACT("Blood Glucose -- metabolism")) OR (EMB.EXACT("glucose blood level"))

S3 ab(hypoglyceamia OR hypoglycemia OR hypoglycemic OR hypoglyceamic)

S4 (RTYPE. EXACT ("Conference Abstract" OR "Editorial" OR "Conference Paper" OR "Note" OR "Short Survey" OR "Letter") OR subt.exact("rat" OR "animal tissue" OR "animal cell" OR "wistar rat" OR "in vitro study"))

S5 S1 AND (S2 OR S3) NOT S4

MESH: thesaurus terms of MEDLINE

EMB: thesaurus terms of EMBASE

MJ: major thesaurus terms

RTYPE: publication type

SUBT: subtitle

“EXPLODE" means searching for a subject term and all its associated narrower terms while “EXACT” means searching for the specified term or phrase only.

"ti" indicates that the descriptor terms in parenthesis exist in the title.

Asterisk and its subsequent number in each bracket (e.g., [*3]) indicate allowing inflections within the number of characters.
