# Supplementary material for: Ability of Current Machine Learning Algorithms to Predict and Detect Hypoglycemia in Patients With Diabetes Mellitus: Meta-analysis
Source: JMIR Diabetes. 2021 Jan 29;6(1):e22458. doi: 10.2196/22458 (PMC7880810; doi:10.2196/22458)
Supplement: Multimedia Appendix 2 [file diabetes_v6i1e22458_app2.docx]

Domain 1: data selection

Risk of bias: Did the selection of patients and data introduce bias?

Signaling question 1: Was a consecutive or random sample of patients enrolled?

Signaling question 2: Was a case-control design avoided?

Signaling question 3: Did the study avoid inappropriate exclusions of patients or datasets?

Applicability: Are there concerns that the included patients and setting do not match the review question?

Domain 2: Index test

Risk of bias: Could the conduct or interpretation of the index test have introduced bias?

Signaling question 1: Were the index test results interpreted without knowledge of the results of the reference standard?

Signaling question 2: If a threshold was used, was it pre-specified?

Applicability: Are there concerns that the index test, its conduct, and/or its interpretation differ from the review question?

Domain 3: Reference standard

Risk of bias: Could the reference standard, its conduct, and/or its interpretation have introduced bias?

Signaling question 1: Were the results of the reference standard interpreted without knowledge of the index test results?

Signaling question 2: If a threshold was used, was it pre-specified?

Applicability: Are there concerns that the target condition as defined by the reference standard does not match the question?

Domain 4: Flow and timing

Risk of bias: Could the patient flow have introduced bias?

Signaling question 1: Was there an appropriate interval between the index test and reference standard?

Signaling question 2: Did all patients receive the same reference standard?

Signaling question 3: Were all patients included in the analysis?
