# Supplementary figures and images for: Ability of Current Machine Learning Algorithms to Predict and Detect Hypoglycemia in Patients With Diabetes Mellitus: Meta-analysis
Source: JMIR Diabetes. 2021 Jan 29;6(1):e22458. doi: 10.2196/22458 (PMC7880810; doi:10.2196/22458)

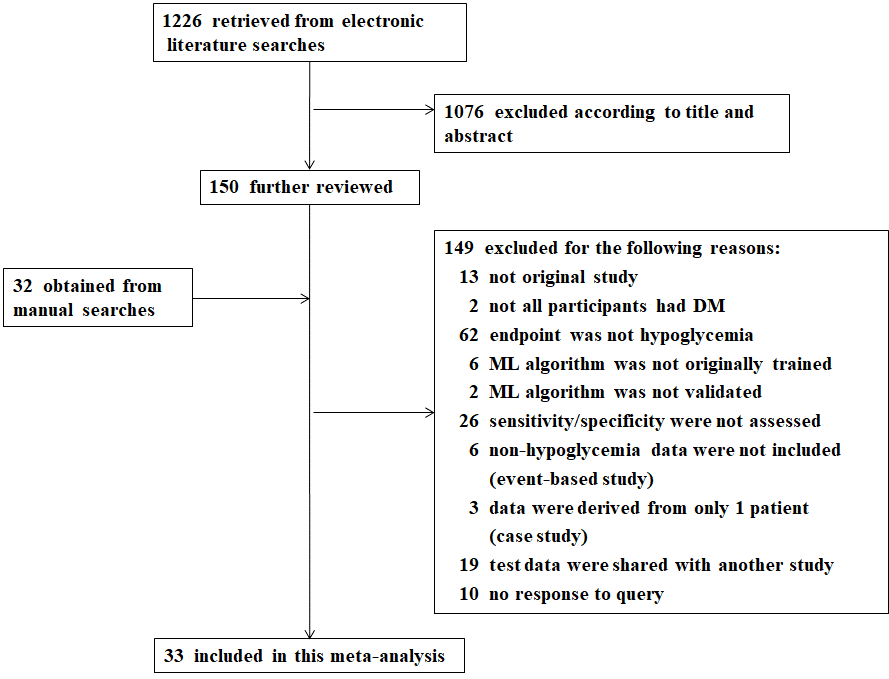


Abbreviations: DM, diabetes mellitus; ML, machine learning

Supplement: Multimedia Appendix 3 [file diabetes_v6i1e22458_app3.docx]
