# Supplementary material for: Ability of Current Machine Learning Algorithms to Predict and Detect Hypoglycemia in Patients With Diabetes Mellitus: Meta-analysis
Source: JMIR Diabetes. 2021 Jan 29;6(1):e22458. doi: 10.2196/22458 (PMC7880810; doi:10.2196/22458)
Supplement: Multimedia Appendix 4 [file diabetes_v6i1e22458_app4.docx]

| Database | ^a^ No. of articles |
| --- | --- |
| 1. EMBASE®‎ | 883 |
| 2. EMCare®‎ | 308 |
| 3. MEDLINE®‎ | 189 |
| 4. SciSearch®: a Cited Reference Science Database‎ | 130 |
| 5. Inspec®‎ | 112 |
| 6. Current Contents® Search‎ | 57 |
| 7. Gale Group Health Periodicals Database‎ | 34 |
| 8. ProQuest Newsstand Professional‎ | 24 |
| 8. ProQuest Dissertations and Theses Professional‎ | 24 |
| 10. Computer and Information Systems Abstracts‎ | 23 |
| 11. Health Research Full Text Professional‎ | 20 |
| 11.ToxFile®‎ | 20 |
| 13. BIOSIS Previews®‎ | 19 |
| 13. Ei Compendex®‎ | 19 |
| 13. ABI/INFORM® Professional Advanced‎ | 19 |
| 16. Biotechnology Research Abstracts‎ | 14 |
| 17. Mechanical & Transportation Engineering Abstracts‎ | 12 |
| 17. Social SciSearch®‎ | 12 |
| 19. Electronics & Communications Abstracts‎ | 11 |
| 20. Immunology Abstracts‎ | 10 |
| 21. Genetics Abstracts‎ | 9 |
| 21. Bacteriology Abstracts (Microbiology B)‎ | 9 |
| 21. Virology and AIDS Abstracts‎ | 9 |
| 21. Algology Mycology and Protozoology Abstracts (Microbiology C)‎ | 9 |
| 21. Nucleic Acids Abstracts‎ | 9 |
| 26. Solid State and Superconductivity Abstracts‎ | 8 |
| 27. Animal Behavior Abstracts‎ | 7 |
| 28. Civil Engineering Abstracts‎ | 6 |
| 28. Neurosciences Abstracts‎ | 6 |
| 30. Meteorological & Geoastrophysical Abstracts‎ | 5 |
| 30. Ecology Abstracts‎ | 5 |
| 30. Entomology Abstracts‎ | 5 |
| 30. Abstracts in New Technology & Engineering‎ | 5 |
| 30. Gale Group Trade & Industry Database™‎ | 5 |
| 35. Calcium & Calcified Tissue Abstracts‎ | 4 |
| 35. Toxicology Abstracts‎ | 4 |
| 35. Materials Business File‎ | 4 |
| 35. COVID-19 Research‎ | 4 |
| 39. Health & Safety Science Abstracts‎ | 3 |
| 39. Aerospace Database‎ | 3 |
| 39. APA PsycInfo®‎ | 3 |
| 39. TOXLINE‎ | 3 |
| 39. Chemical Engineering & Biotechnology Abstracts‎ | 3 |
| 39. Gale Group Computer Database™‎ | 3 |
| 39. Global Health‎ | 3 |
| 46. CAB ABSTRACTS‎ | 2 |
| 46. Engineered Materials Abstracts‎ | 2 |
| 46. Aluminium Industry Abstracts‎ | 2 |
| 46. Corrosion Abstracts‎ | 2 |
| 46. Ceramic Abstracts‎ | 2 |
| 46. METADEX‎ | 2 |
| 46. British Nursing Index‎ | 2 |
| 53. Derwent Drug File‎ | 1 |
| 53. Gale Group PROMT®‎ | 1 |
| 53. AGRICOLA‎ | 1 |
| 53. Chemoreception Abstracts‎ | 1 |
| 53. Copper Technical Reference Library‎ | 1 |
| 53. Industrial and Applied Microbiology Abstracts (Microbiology A)‎ | 1 |
| 53. Oncogenes and Growth Factors Abstracts‎ | 1 |
| 53.International Pharmaceutical Abstracts‎ | 1 |
| 53. BIOSIS® Toxicology | 1 |

^a^ Total number of retrieved articles was 1226, which is inconsistent with the sum of the number of articles published in each database because some retrieved articles were published by two or more databases.
