# Supplementary material for: Ability of Current Machine Learning Algorithms to Predict and Detect Hypoglycemia in Patients With Diabetes Mellitus: Meta-analysis
Source: JMIR Diabetes. 2021 Jan 29;6(1):e22458. doi: 10.2196/22458 (PMC7880810; doi:10.2196/22458)
Supplement: Multimedia Appendix 5 [file diabetes_v6i1e22458_app5.docx]

| study source | evalu- |  |
| --- | --- | --- |
|  | -ation^a^ |  |
| Bertachi (2020)[26] | Pre | CGM, insulin, meal |
| Dave (2020)[27] | Pre | CGM, insulin, CHO, age, gender, A1C |
| Elhadd (2020)[28] | Pre | CGM, PA, height, BW, A1C, lipid, renal, BP |
| Marcus (2020)[29] | Pre | CGM |
| Mosquera-Lopez | Pre | CGM, insulin, meal |
| (2020)[30] |  |  |
| Mueller (2020)[31] | Pre | 388 variables consisting of demographics, PH, medication, and lab. profiles |
| Ngo (2020)[32] | Dec | EEG |
| Ruan (2020)[33] | Pre | 42 variables consisting of demographics, vital signs, Lab. and |
|  |  | medication profiles |
| Rubega (2020)[25] | Dec | EEG |
| Chen (2019)[34] | Dec | messages |
| Guemes (2019)[35] | Pre | CGM, insulin, meal, past history of hypoglycemic events |
| Jensen (2019)[36] | Pre | CGM, BMI |
| Jin (2019)[37] | Dec | health records |
| Oviedo (2019)[38] | Pre | CGM, insulin, CHO |
| Reddy (2019)[39] | Pre | insulin, glucagon, gender, BW, height, BMI, BG-start^b^, ExI |
| Seo (2019)[40] | Pre | CGM |
| Arthur (2018)[41] | Pre | CGM, insulin, CHO, PA |
| Toffanin (2018)[42] | Pre | CGM, meal, insulin |
| Ling (2016)[43] | Dec | ECG |
| Sampath (2016)[44] | Pre | IGM |
| Sudharsan (2015)[45] | Pre | IGM, glucose-lowering medications |
| Eljil (2014)[46] | Pre | CGM |
| Plis (2014)[47] | Pre | CGM, meal, insulin |
| Jensen (2013)[48] | Dec | CGM, insulin |
| Nguyen (2013)[49] | Dec | EEG |
| Nguyen (2012)[50] | Dec | EEG |
| Nuryani (2012)[51] | Dec | ECG |
| Chan (2011)[15] | Dec | ECG |
| Ling (2010)[52] | Dec | EEG |
| Nguyen (2010)[20] | Dec | EEG |
| Skladnev (2010)[53] | Dec | ECG, skin |
| Zhang (2008)[54] | Pre | IGM, insulin |
| Iaione (2005)[55] | Dec | EEG |

Abbreviations: A1C, glycohemoglobin A1c; BMI, body mass index; BW, body weight; CGM, continuous glucose monitoring; CHO, carbohydrate intake; Dec, detecting hypoglycemia; ECG, electrocardiography; EEG, electroencephalogram; ExI, exercise intensity; IGM, intermittent glucose monitoring; Lab., laboratory data; PA, physical activity; PH, past history of diseases; Pre, predicting hypoglycemia

^a^ evaluation for model performance in detecting or predicting hypoglycemia

^b^ blood glucose level at the start of exercise
