# Supplementary material for: Ability of Current Machine Learning Algorithms to Predict and Detect Hypoglycemia in Patients With Diabetes Mellitus: Meta-analysis
Source: JMIR Diabetes. 2021 Jan 29;6(1):e22458. doi: 10.2196/22458 (PMC7880810; doi:10.2196/22458)
Supplement: Multimedia Appendix 6 [file diabetes_v6i1e22458_app6.docx]

| study source | item^a^ | ROB |  |  |  |  |  |  |  |  |  |  |  |  |  | App |  |  |  |
| --- | --- | --- | --- | --- | --- | --- | --- | --- | --- | --- | --- | --- | --- | --- | --- | --- | --- | --- | --- |
|  |  | D1 |  |  |  | D2 |  |  | D3 |  |  | D4 |  |  |  | D1 | D2 | D3 | total |
|  |  | score | SQ1 | SQ2 | SQ3 | score | SQ1 | SQ2 | score | SQ1 | SQ2 | score | SQ1 | SQ2 | SQ3 | score | score | score |  |
| Bertachi (2020)[26] | Pre | 1 | Y | Y | Y | 0 | Y | N | 1 | Y | Y | 1 | Y | Y | Y | 1 | 1 | 0 | 5 |
| Dave (2020)[27] | Pre | 1 | Y | Y | Y | 1 | Y | Y | 1 | Y | Y | 1 | Y | Y | N | 1 | 1 | 0 | 6 |
| Elhadd (2020)[28] | Pre | 1 | Y | Y | Y | 0 | Y | N | 0 | Y | N | 0 | Y | N | Y | 1 | 1 | 0 | 3 |
| Marcus (2020)[29] | Pre | 1 | Y | Y | Y | 1 | Y | Y | 1 | Y | Y | 1 | Y | Y | Y | 1 | 1 | 0 | 6 |
| Mosquera-Lopez (2020)[30] | Pre | 1 | Y | Y | Y | 1 | Y | Y | 1 | Y | Y | 1 | Y | Y | Y | 1 | 1 | 0 | 6 |
| Mueller (2020)[31] | Pre | 1 | Y | Y | Y | 0 | N | Y | 1 | Y | Y | 1 | Y | Y | Y | 1 | 1 | 1 | 6 |
| Ngo (2020)[32] | Dec | 1 | Y | Y | Y | 1 | Y | Y | 1 | Y | Y | 1 | Y | Y | Y | 1 | 1 | 1 | 7 |
| Ruan (2020)[33] | Pre | 1 | Y | Y | Y | 0 | Y | N | 1 | Y | Y | 0 | Y | Y | N | 1 | 1 | 1 | 5 |
| Rubega (2020)[25] | Dec | 0 | Y | N | Y | 0 | N | Y | 1 | Y | Y | 1 | Y | Y | Y | 0 | 1 | 1 | 4 |
| Chen (2019)[34] | Dec | 1 | Y | Y | Y | 0 | Y | N | 0 | Y | N | 1 | Y | Y | Y | 1 | 1 | 0 | 4 |
| Guemes (2019)[35] | Pre | 1 | Y | Y | Y | 1 | Y | Y | 1 | Y | Y | 1 | Y | Y | Y | 1 | 1 | 0 | 6 |
| Jensen (2019)[36] | Pre | 1 | Y | Y | Y | 1 | Y | Y | 1 | Y | Y | 1 | Y | Y | Y | 1 | 1 | 1 | 7 |
| Jin (2019)[37] | Dec | 1 | Y | Y | Y | 0 | Y | N | 1 | Y | Y | 1 | Y | Y | Y | 1 | 1 | 1 | 6 |
| Oviedo (2019)[38] | Pre | 1 | Y | Y | Y | 0 | N | Y | 1 | Y | Y | 1 | Y | Y | Y | 1 | 1 | 0 | 5 |
| Reddy (2019)[39] | Pre | 1 | Y | Y | Y | 1 | Y | Y | 1 | Y | Y | 1 | Y | Y | Y | 1 | 1 | 1 | 7 |
| Seo (2019)[40] | Pre | 1 | Y | Y | Y | 0 | Y | N | 1 | Y | Y | 1 | Y | Y | Y | 1 | 1 | 0 | 5 |
| Arthur (2018)[41] | Pre | 1 | Y | Y | Y | 1 | Y | Y | 1 | Y | Y | 1 | Y | Y | Y | 1 | 1 | 0 | 6 |
| Toffanin (2018)[42] | Pre | 1 | Y | Y | Y | 1 | Y | Y | 1 | Y | Y | 1 | Y | Y | Y | 1 | 1 | 0 | 6 |
| Ling (2016)[43] | Dec | 1 | Y | Y | Y | 1 | Y | Y | 1 | Y | Y | 1 | Y | Y | Y | 1 | 1 | 0 | 6 |
| Sampath (2016)[44] | Pre | 1 | Y | Y | Y | 1 | Y | Y | 1 | Y | Y | 1 | Y | Y | Y | 1 | 1 | 1 | 7 |
| Tkachenko (2016)[63] | Pre | 1 | Y | Y | Y | 1 | Y | Y | 1 | Y | Y | 1 | Y | Y | Y | 1 | 1 | 0 | 6 |
| Sudharsan (2015)[45] | Pre | 1 | Y | Y | Y | 1 | Y | Y | 1 | Y | Y | 1 | Y | Y | Y | 1 | 1 | 1 | 7 |
| Eljil (2014)[46] | Pre | 0 | Y | Y | N | 0 | Y | N | 1 | Y | Y | 1 | Y | Y | Y | 1 | 1 | 0 | 4 |
| Plis (2014)[47] | Pre | 1 | Y | Y | Y | 1 | Y | Y | 1 | Y | Y | 1 | Y | Y | Y | 1 | 1 | 0 | 6 |
| Jensen (2013)[48] | Dec | 1 | Y | Y | Y | 0 | Y | N | 1 | Y | Y | 1 | Y | Y | Y | 1 | 1 | 1 | 6 |
| Nguyen (2013)[49] | Dec | 1 | Y | Y | Y | 1 | Y | Y | 1 | Y | Y | 1 | Y | Y | Y | 1 | 1 | 0 | 6 |
| Nguyen (2012)[50] | Dec | 1 | Y | Y | Y | 1 | Y | Y | 1 | Y | Y | 1 | Y | Y | Y | 1 | 1 | 0 | 6 |
| Nuryani (2012)[51] | Dec | 1 | Y | Y | Y | 1 | Y | Y | 0 | Y | N | 1 | Y | Y | Y | 1 | 0 | 0 | 4 |
| Chan (2011)[15] | Dec | 1 | Y | Y | Y | 1 | Y | Y | 1 | Y | Y | 1 | Y | Y | Y | 1 | 1 | 0 | 6 |
| Ling (2010)[52] | Dec | 1 | Y | Y | Y | 1 | Y | Y | 1 | Y | Y | 1 | Y | Y | Y | 1 | 1 | 0 | 6 |
| Nguyen (2010)[20] | Dec | 1 | Y | Y | Y | 1 | Y | Y | 1 | Y | Y | 1 | Y | Y | Y | 1 | 1 | 1 | 7 |
| Skladnev (2010)[53] | Dec | 1 | Y | Y | Y | 1 | Y | Y | 1 | Y | Y | 1 | Y | Y | Y | 1 | 1 | 1 | 7 |
| Zhang (2008)[54] | Pre | 1 | Y | Y | Y | 0 | N | Y | 1 | Y | Y | 1 | Y | Y | Y | 1 | 1 | 0 | 5 |
| Iaione (2005)[55] | Dec | 0 | Y | N | Y | 0 | N | Y | 1 | Y | Y | 0 | N | Y | Y | 0 | 1 | 1 | 3 |

^a^ evaluation item for model performance (i.e., ability to detect hypoglycemia or ability to predict hypoglycemia)

Abbreviations: App, applicability; Dec, detective ability for hypoglycemia; N, No; Pre, predictive ability for hypoglycemia; ROB, risk of bias; Y, Yes
